# Aptamer-Functionalized Fluorescent Silica Nanoparticles for Highly Sensitive Detection of Leukemia Cells

**DOI:** 10.1186/s11671-016-1512-8

**Published:** 2016-06-14

**Authors:** Juntao Tan, Nuo Yang, Zixi Hu, Jing Su, Jianhong Zhong, Yang Yang, Yating Yu, Jianmeng Zhu, Dabin Xue, Yingying Huang, Zongqiang Lai, Yong Huang, Xiaoling Lu, Yongxiang Zhao

**Affiliations:** 1 National Center for International Research of Biological Targeting Diagnosis and Therapy, Guangxi Key Laboratory of Biological Targeting Diagnosis and Therapy Research, Collaborative Innovation Center for Targeting Tumor Diagnosis and Therapy, Guangxi Medical University, Nanning, Guangxi 530021 China; 2 Department of Oncologic Surgery, Affiliated Tumor Hospital of Guangxi Medical University, Nanning, Guangxi China; 3 Department of Hematology, The First Affiliated Hospital of Guangxi Medical University, Nanning, Guangxi China; 4 Department of Thoracic Surgery, Affiliated Tumor Hospital of Guangxi Medical University, Nanning, Guangxi China

**Keywords:** Ramos Cell, Detect Cancer Cell, Reverse Microemulsion Method, Human Acute Lymphoblastic Leukemia, Amide Coupling

## Abstract

A simple, highly sensitive method to detect leukemia cells has been developed based on aptamer-modified fluorescent silica nanoparticles (FSNPs). In this strategy, the amine-labeled Sgc8 aptamer was conjugated to carboxyl-modified FSNPs via amide coupling between amino and carboxyl groups. Sensitivity and specificity of Sgc8-FSNPs were assessed using flow cytometry and fluorescence microscopy. These results showed that Sgc8-FSNPs detected leukemia cells with high sensitivity and specificity. Aptamer-modified FSNPs hold promise for sensitive and specific detection of leukemia cells. Changing the aptamer may allow the FSNPs to detect other types of cancer cells.

## Background

Leukemia is a common, aggressive cancer that seriously affects blood cells, the lymphatic system, and the bone marrow [1, 2]. It seriously interferes with daily activities and increases the risk of death [3]. Immediate treatment is critical for improving the survival rate of patients with leukemia [4], highlighting the need for early diagnosis using sensitive and specific methods.

Current methods to detect leukemia cells usually rely on various cytochemical analyses of peripheral blood cells and bone marrow, including karyotyping [5], flow cytometry-based immunophenotyping [6], microarrays [7], cell enrichment [8], polymerase chain reaction [9], and electrochemical sensors [10]. These methods have various limitations, such as low sensitivity, high cost, and high complexity. Thus, a simple, highly sensitive method is urgently needed to detect leukemia cells.

A technology that shows promise in this regard is fluorescent silica nanoparticles (FSNPs), which are superior to organic dye probes [11]. FSNPs easily undergo surface modifications, and they show good biocompatibility [12]. On the other hand, FSNPs by themselves are not selective for cancerous tissue, so researchers have turned to aptamers to confer specificity and selectivity on FSNPs biodistribution and activity.

Aptamers are single-stranded nucleic acids selected from random-sequence libraries of DNA or RNA through an in vitro selection process called systematic evolution of ligands by exponential enrichment (SELEX) [13]. The resulting sequences can bind a wide range of targets with high affinity, including small molecules [14, 15], proteins [16], and even whole cells [17]. Numerous studies have shown that aptamer-functionalized FSNPs can target many types of cancer [18–20]. These results suggest that aptamer-FSNPs may allow reliable identification of leukemia cells.

Here, we design and prepare FSNPs modified with the aptamer Sgc8, which binds tightly and specifically to T acute lymphocytic leukemia cells. The resulting Sgc8-FSNPs were analyzed by dynamic light scattering, transmission electron microscopy (TEM), zeta potential measurement, Fourier transform infrared (FT-IR) spectroscopy, ultraviolet–visible (UV–vis) spectroscopy, and fluorimetry. Their ability to detect leukemia cells was assessed using flow cytometry and fluorescence microscopy. Their toxicity was evaluated in vitro and in vivo using the CKK-8 assay and histology of organ sections. Our results suggest that this novel probe may be a reliable and efficient way to detect leukemia cells.

## Methods

### Materials

Fluorescein isothiocyanate (FITC), 3-aminopropylmethyldimethoxysilane (APTMS), Triton X-100, *n*-hexanol, cyclohexane, tetraethyl orthosilicate (TEOS), ammonium hydroxide (NH_4_OH, 25-28 wt %), 1-ethyl-3-(3-dimethylaminopropyl)carbodiimide hydrochloride (EDC), *N*-hydroxysulfosuccinimide sodium salt (sulfo-NHS), and *N*-[(3-trimethoxysilyl)propyl]ethylenediamine triacetic acid trisodium salt (TMS-EDTA) were bought from Sigma Chemical (St. Louis, MO, USA). DNA oligonucleotides were synthesized by Shanghai Sangon Biological Engineering Technology & Services (Shanghai, China). These oligonucleotides included the amine-labeled Sgc8 aptamer (Sgc8), 5′-NH_2_-TTTTTTTTTTATCTAACTGCTGCGCCGCCGGGAAAATACTGTACGGTTAGA-3′; and amine- and FITC-labeled Sgc8 (FITC-Sgc8), 5′-NH_2_-TTTTTTTTTTATCTAACTGCTGCGCCGCCGGGAAAATACTGTACGGTTAGA-FITC-3′.

### Preparation of Sgc8-FSNPs

A mixture of FITC in anhydrous ethanol (1 mg/l, 1 ml) and APTMS (10 μl) was stirred for 24 h to prepare FITC-APTMS. FSNPs were prepared as a water-in-oil microemulsion in a solution containing 7.5 ml of cyclohexane, 1.6 ml of *n*-hexanol, 1.77 ml of Triton X-100, and 500 μl of distilled water. This solution was stirred for 30 min at room temperature, and then 150 μl of FITC-APTMS, 100 μl of TEOS, and 60 μl of NH_4_OH were successively added, after which the solution was stirred for another 24 h. Then 30 μl of NTTS was added, and the mixture was stirred for another 24 h. The resulting carboxyl-modified FSNPs (FSNPs-COOH) were precipitated in an equal volume of acetone and collected by alternately centrifuging and washing three times with deionized water and fresh 95 % ethanol.

Amine-labeled Sgc8 was cross-linked to FSNPs-COOH using EDC as follows. Briefly, FSNPs-COOH (ca. 0.1 mg, 1 ml) were washed in MES buffer (0.1 M, pH 5.65) and resuspended in 950 μl of MES buffer (0.1 M, pH 5.65). This solution was added with 50 μl of 10 μM amine-labeled Sgc8, 1.8 mg of EDC, and 3.5 mg of sulfo-NHS, after which the mixture was shaken gently for 3 h at room temperature. The nanoparticles were washed three times with PBS (0.1 M, pH 7.4) and incubated with 0.05 % BSA in PBS for 1 h at room temperature to block free carboxyl groups. Sgc8-FSNPs were collected by three rounds of centrifugation and resuspension in PBS and then stored at 4 °C in MES buffer (0.1 M, pH 5.65) containing 1 % BSA until use.

### Characterization of Sgc8-FSNPs

The structural features of Sgc8-FSNPs were analyzed using TEM (H-7650, Japan), while average size and zeta potential were determined using dynamic light scattering on a Zetasizer Nano instrument (Malvern Instruments, UK). The modification of surface carboxyl and amino groups on FSNPs was confirmed using FT-IR spectroscopy (Nicolet-5700, USA). The UV–vis absorption spectrum was obtained (UV 2550, Shimadzu, Japan), as well as fluorescence emission spectrum (Hitachi F-4600, Japan). For all these measurements, a concentration of 0.1 mg/ml Sgc8-FSNPs was used.

The number of moles of FSNPs was calculated using the method of Pang et al. [12], and the average number of aptamers on the nanoparticle surface was estimated based on fluorescence signal as follows. Briefly, initial fluorescence intensity (*I*_1_) was measured immediately after mixing FITC-Sgc8 with FSNPs-COOH. When amide coupling of FITC-Sgc8 to FSNPs-COOH via the carboxyl and amino groups was complete, the supernatant was washed to remove unbound FITC-Sgc8, and the fluorescence intensity (*I*_2_) of the resuspension was measured. The ratio *I*_2_/*I*_1_ was taken to indicate the efficiency of aptamer modification on the surface of FSNPs. Then the amount of Sgc8 on FSNPs was calculated by taking the actual number of FITC-Sgc8 molecules on FSNPs and dividing by the number of FSNPs.

### Cells and Animals

Cell lines were obtained from the National Center for International Research of Biological Targeting Diagnosis and Therapy of Guangxi Medical University. CCRF-CEM cells (human acute lymphoblastic leukemia, T cell) as a positive control for leukemia cells and Ramos cells (human Burkitt’s lymphoma, B cell) as a negative control were cultured in 1640 medium supplemented with 10 % fetal bovine serum (FBS, Hyclone) and 100 U/ml penicillin-streptomycin. Both cell lines were incubated with Sgc8-FSNPs. In a separate experiment, L-02 and 293T cells were incubated with Sgc8-FSNPs to evaluate their cytotoxicity. These two cell lines were cultured in DMEM medium supplemented with 10 % FBS and 100 U/ml penicillin-streptomycin.

Six-week-old female BALB/c nude mice from the Guangxi Laboratory Animal Center (Guangxi, China) were raised in sterile conditions in a laminar flow hood. All protocols were conducted in accordance with the guidelines of the Animal Ethics Committee of Guangxi Medical University, Nanning, Guangxi, China.

### Flow Cytometry

Cells were cultured at a density of 3.0 × 10^5^ cells/ml, collected by centrifugation, and resuspended in 200 μl of PBS. Then cells were incubated on ice for 30 min in the dark with Sgc8-FSNPs, FSNPs, or FITC-Sgc8 in 200 μl of cell culture medium or binding buffer (PBS supplemented with 5 mM MgCl_2_, 4.5 g/l glucose, and 1 mg/ml BSA). Cells were washed with PBS, suspended in 200 μl of binding buffer and analyzed by flow cytometry (Beckman Coulter Epics XL, USA). Data were analyzed using EXPO32 ADC Analysis software.

### Cell Imaging

CCRF-CEM and Ramos cells were cultured for 12 h in six-well dishes at a density of 3 × 10^5^ cells per well. Cells were washed three times with PBS and incubated with Sgc8-FSNPs, FSNPs, or FITC-Sgc8, respectively, in binding buffer on ice for 30 min in the dark. Cells were washed three times with PBS, fixed with 4 % polyoxymethylene (Sigma-Aldrich) for 20 min, and stained with 4′, 6-diamidino-2-phenylindole dihydrochloride (DAPI; Life Co., USA) for 5 min in the dark. Finally, cells were washed three times with PBS and examined by fluorescence microscopy (Nikon DS-Ri1, Japan).

### In Vitro and In Vivo Toxicity of Sgc8-FSNPs

Two types of normal cells, such as L-02 and 293T cells, were cultured for 24 h in 96-well plates at an initial density of 1.0 × 10^4^ cells/well and then exposed to Sgc8-FSNPs at 0.1, 0.5, or 1.0 mg/ml for 8, 16, and 24 h. Cytotoxicity of the treatments was assessed using the CCK-8 assay [21]. At the specific time points, the culture medium was removed and cells were washed three times with PBS. Then 10 μl of CCK-8 solution was added to each well in 90 μl of serum-free DMEM medium. After incubation for 4 h at 37 °C, the absorbance of each well at 450 nm was measured using a Bio-Rad microplate reader (DTX880, Beckman Coulter, USA).

To examine the in vivo toxicity of Sgc8-FSNPs, nude mice received a single tail vein injection of 200 μl of PBS (control group) or PBS containing Sgc8-FSNPs, FSNPs, or FITC-Sgc8 (1 mg/ml). At 1 week after injection, mice were sacrificed, and sections of major tissues (heart, lung, liver, spleen, kidney) were immersed in 10 % formaldehyde solution, dehydrated, and paraffin-embedded. Serial sections (4 μm) were cut and stained with hematoxylin-eosin.

### Statistical Analyses

Each experiment was carried out in triplicate. Data were expressed as mean ± SD or as median (range). All statistical analyses were performed using GraphPad Prism (GraphPad Software, San Diego, CA, USA). *P* < 0.05 was considered the threshold of significance in all analyses.

## Results and Discussion

**Fig. 1:**
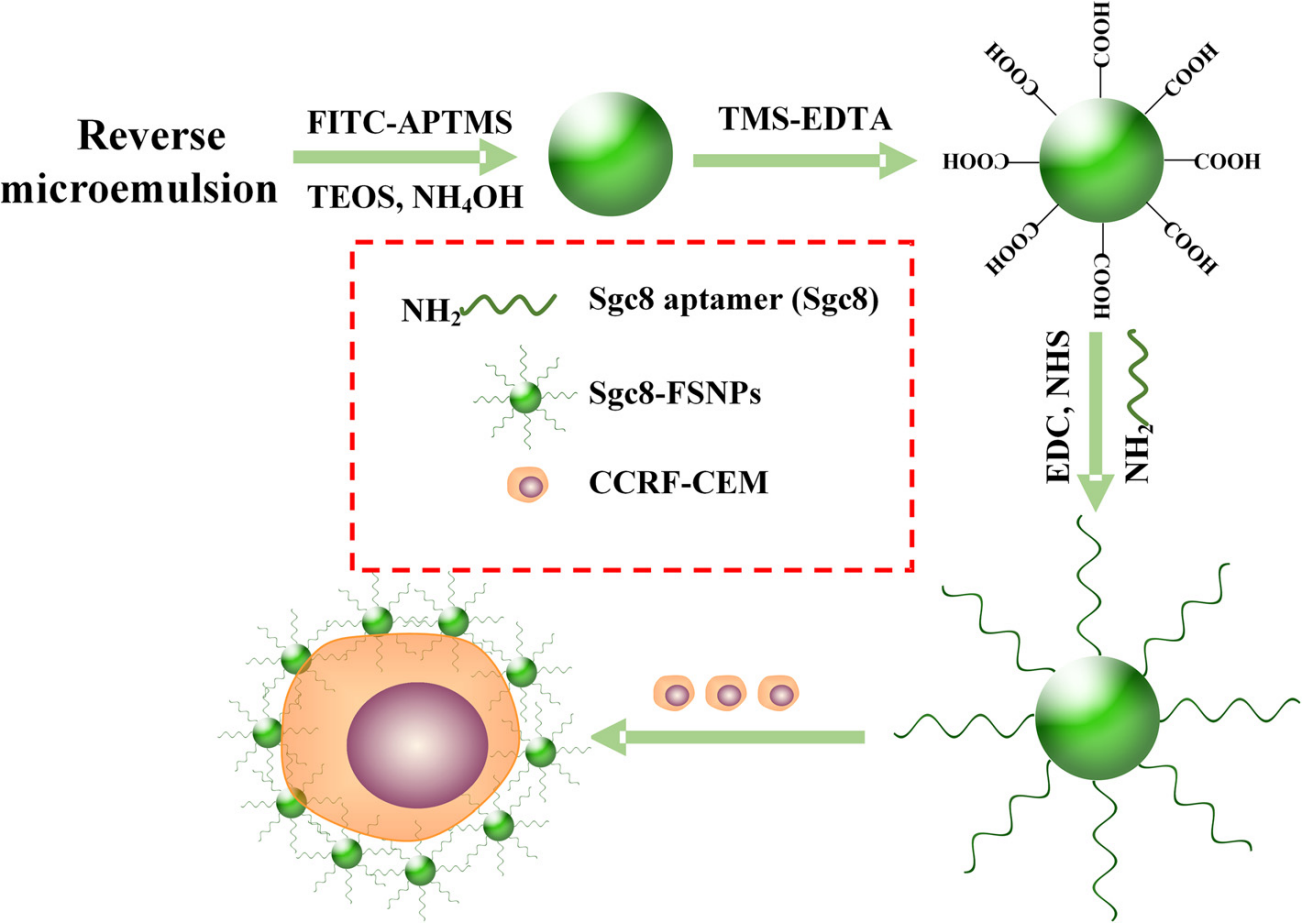
Schematic illustration of Sgc8 aptamer-modified FSNPs for highly sensitive detection of leukemia cells

In this present study, we combined the Sgc8 aptamer, developed through live-cell SELEX to bind tightly and specifically to CCRF-CEM cells [22], with FSNPs as a signal reporter in order to develop a novel detection probe for leukemia (Fig. 1). The combination of specific targeting by Sgc8 and the strong fluorescence, photostability, and biocompatibility of FSNPs [11] may make Sgc8-FSNPs a clinically useful reagent in the fight to diagnose leukemia as early as possible to ensure timely treatment.

### Characterization of Sgc8-FSNPs

**Fig. 2:**
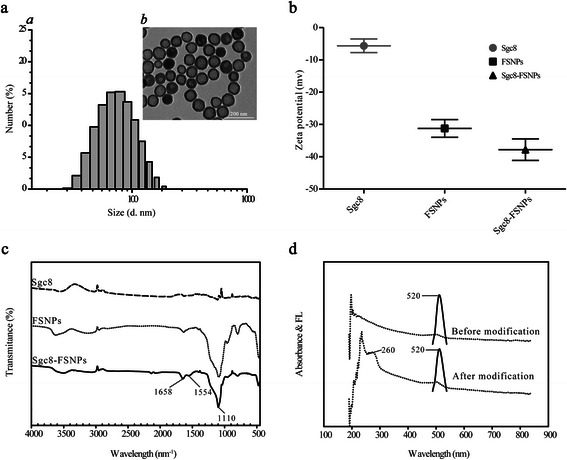
Characterization of Sgc8-FSNPs. **a** Particle size distribution of *a* Sgc8-FSNPs based on dynamic light scattering. *b* TEM of Sgc8-FSNPs. **b** Zeta potential of Sgc8, FSNPs, and Sgc8-FSNPs. **c** FT-IR spectra of Sgc8, FSNPs, and Sgc8-FSNPs. **d** UV–vis absorbance (*solid line*) and fluorescence emission spectra (*dashed line*) of FSNPs before and after modification with Sgc8

The reverse microemulsion method is widely used to prepare silica nanoparticles, because it can yield uniform, monodisperse particles [23], and our FSNPs were indeed uniform in shape and size (Fig. 2a, panels *a* and *b*). Average diameter was 70.52 ± 2.64 nm for FSNPs and 75.18 ± 2.89 nm for Sgc8-FSNPs; the corresponding zeta potentials were −31.21 ± 2.75 mV and −37.57 ± 3.33 mV (Table 1). The more negative zeta potential for Sgc8-FSNPs than FSNPs (Fig. 2b) likely reflects the additional negative charge of the phosphate groups on the DNA aptamer [24]. The efficiency of aptamer conjugation onto the surface of FSNPs was determined indirectly from the change in fluorescence intensity during the reaction between FITC-Sgc8 and SNPs. Based on the conversion that 1 mg of SNPs with an average diameter of about 60 nm is approximately 6.8 pmol [12], and assuming that the initial number of aptamers was 500 pmol and the conjugation efficiency was 43 %, we estimated that the surface of our FSNPs contained approximately 420 aptamers (Table 1).

**Table 1:** Size, zeta potential, and surface charge characteristics of FSNPs and Sgc8-FSNPs

Sample	Size (nm)	Zeta (mV)	Aptamer-FSNPs
FSNPs	70.52 ± 2.64	−31.21 ± 2.75	–
Sgc8-FSNPs	75.18 ± 2.89	−37.57 ± 3.33	419.92 ± 39.83

The FT-IR spectra of Sgc8, FSNPs, and Sgc8-FSNPs are shown in Fig. 2c. The spectrum for Sgc8-FSNPs showed peaks at 1658 cm^−1^ (C=O carbonyl stretching vibrations), 1554 cm^−1^ (C–N stretching vibrations and N–H bending), and 1100 cm^−1^ (Si–O stretching vibrations). The feature of amides II was observed around 1554 cm^−1^ originated from C–N stretching vibrations and N–H bending [25]. The peak at 1554 cm^−1^ was observed at the spectrum for Sgc8-FSNPs, so it likely reflects the attachment of the amine-labeled aptamer Sgc8 to the surface of FSNPs-COOH.

Figure 2d shows the UV–vis absorbance spectrum (solid line) and fluorescence emission spectrum (dashed line) of FSNPs before and after aptamer conjugation. The UV–vis spectrum of Sgc8-FSNPs showed a DNA absorbance peak at 260 nm that was absent from the spectrum of FSNPs, suggesting that the aptamer had indeed conjugated with the nanoparticles. The fluorescence emission spectra of both FSNPs and Sgc8-FSNPs showed a peak at 520 nm, corresponding to FITC. This indicates that aptamer addition does not change the fluorescence properties of FSNPs [26].

These results of characterization of Sgc8-FSNPs indicate that we successfully conjugated aptamer to the surface of FSNPs.

### Flow Cytometric Detection of Leukemia Cells

**Fig. 3:**
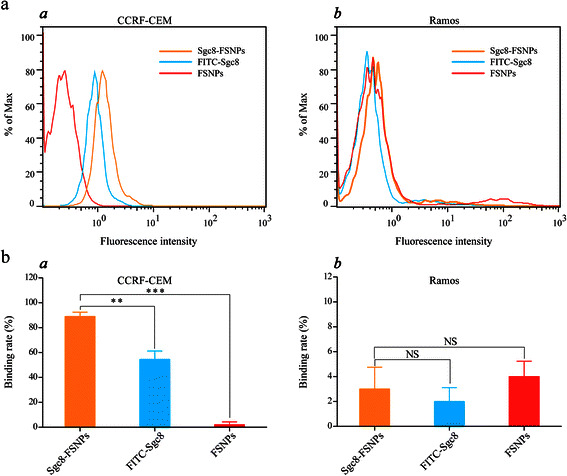
Flow cytometric detection of leukemia cells. **a** Sgc8-FSNPs, FITC-Sgc8, or FSNPs were mixed with *a* CCRF-CEM cells or *b* Ramos cells. **b** Statistical graph for rates of binding to *a* CCRF-CEM cells and *b* Ramos cells by Sgc8-FSNPs, FITC-Sgc8, or FSNPs. *NS*, not significant. **P < 0.01, ***P < 0.001

At first, the ability of Sgc8-FSNPs to detect leukemia cells was investigated by flow cytometry. Ramos cells were used as negative cells, and FSNPs were used as control probe. For the detection of CCRF-CEM cells, stronger fluorescence intensity was observed on Sgc8-FSNPs than FITC-Sgc8, while no fluorescence signal was found on FSNPs (Fig. 3a, panel *a*). In addition, no obvious fluorescence signal was observed on Ramos cells after incubation with Sgc8-FSNPs, FITC-Sgc8, and FSNPs, respectively (Fig. 3a, panel *b*). Similar results were obtained by statistical graph of the binding rate of CEM and Ramos cells (Fig. 3b, panels *a* and *b*). These results suggested that Sgc8-FSNPs can be used to detect CEM cells with higher sensitivity than FITC-Sgc8.

### Fluorescence Microscopy

**Fig. 4:**
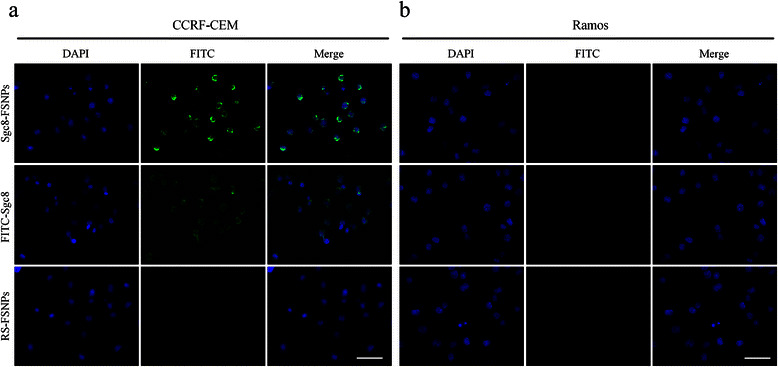
Fluorescence micrographs of CCRF-CEM and Ramos cells after mixing with Sgc8-FSNPs, FITC-Sgc8, or FSNPs. **a** CCRF-CEM cells after incubation. **b** Ramos cells after incubation. Nuclei were stained with DAPI and examined in the blue channel, while FSNPs and FITC were examined in the green channel. The blue and green channels were combined in the merged images

To confirm whether the synthesized Sgc8-FSNPs can recognize and bind CEM cells, fluorescence microscopy images were performed with the same brightness and contrast. As shown in Fig. 4a, both Sgc8-FSNPs and FITC-Sgc8 stained surface of CCRF-CEM cells with green fluorescence, while FSNPs did not. Stronger green fluorescence was also observed on Sgc8-FSNPs than FITC-Sgc8 (Fig. 4a). Consistent with the analysis of flow cytometry, no green fluorescence was found on the surface of Ramos cells after incubation with Sgc8-FSNPs, FITC-Sgc8, and FSNPs, respectively (Fig. 4b). So, we can agree that Sgc8-FSNPs specially recognize and bind CEM with high sensitivity, which is attributed to the interaction between aptamer and its targeting cells rather than the nonspecific binding between FSNPs and cells. In addition, the fluorescence signal of Sgc8-FSNPs is stronger than FITC-Sgc8, which is caused by fluorescent signal amplification of FSNPs.

### Photostability of Sgc8-FSNPs and FITC-Sgc8

**Fig. 5:**
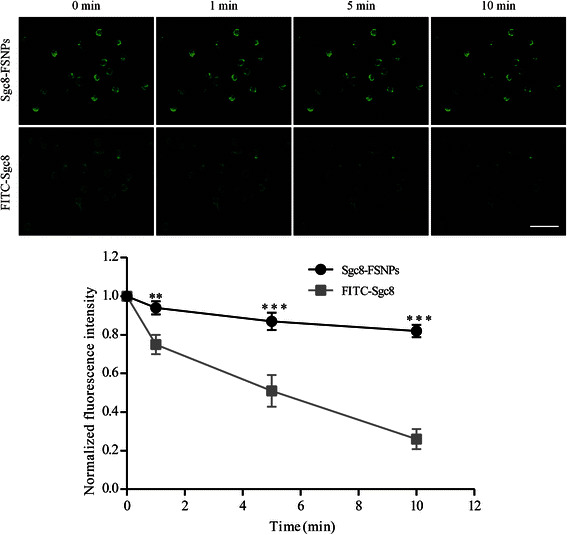
Fluorescence images and quantitation of photostability of Sgc8-FSNPs and FITC-Sgc8

To confirm the anti-photobleaching property of FITC dyes doped inside the silica matrix, fluorescence microscopy was also used to assess the photostability of Sgc8-FSNPs and FITC-Sgc8. Samples were illuminated continuously with a 488-nm laser for 10 min, and fluorescent images were taken at 0, 1, 5, and 10 min. Fluorescence intensity was quantitated using Image Pro (Media Cybernetics, Bethesda, MD, USA). Fluorescence intensity was stronger for Sgc8-FSNPs than for FITC-Sgc8. Normalized fluorescence intensity was also compared between Sgc8-FSNPs and FITC-Sgc8. Intense irradiation for 10 min reduced the fluorescence intensity of Sgc8-FSNPs by only 18 %, while the fluorescence intensity of FITC-Sgc8 fell by 74 %. This suggests that Sgc8-FSNPs were more photostable than FITC-Sgc8. The reason may be explained by FITC molecules well doped the silica shell that separate potential quenching substance from dye molecules through electrostatic interaction [12, 27–29] (Fig. 5).

### In Vitro and In Vivo Toxicity of Sgc8-FSNPs

**Fig. 6:**
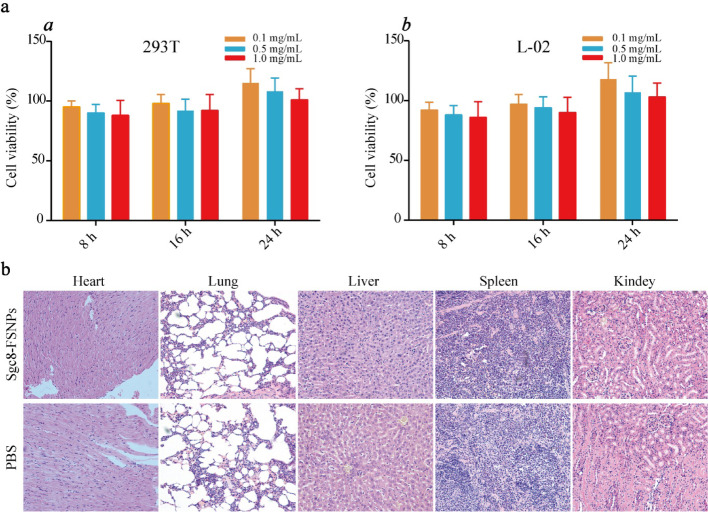
Cell and tissue toxicity tests of Sgc8-FSNPs. ***a*** *a* 293T and *b* L-02 cells were treated with various concentrations of Sgc8-FSNPs, and cell viability was measured every 8 h. ***b*** Nude mice were treated with PBS or Sgc8-FSNPs, and sections from major organs were stained with hematoxylin-eosin and examined by light microscopy

In vitro cytotoxicity of Sgc8-FSNPs was evaluated in the normal cell lines 293T and L-02. Both cell lines showed high viability, based on the CCK-8 assay, after incubation with various concentrations of Sgc8-FSNPs (Fig. 6a, panels *a* and *b*). This suggests that Sgc8-FSNPs show minimal cytotoxicity. We tested this in vivo by treating nude mice with Sgc8-FSNPs and then examining tissue sections from major organs after staining with hematoxylin-eosin. No obvious signs of necrosis or inflammation were observed, confirming that Sgc8-FSNPs show minimal toxic effects (Fig. 6b). This justifies further animal and potentially human testing of Sgc8-FSNPs as a diagnostic probe in the clinic. However, FSNPs show short half-life in the circulatory system, the aptamer is vulnerable to degradation, and the release of fluorescent dye into the blood may increase the risk of systemic toxicity [18]. So, further studies should minimize these limitations for in vivo biological applications.

Our work adds to a growing literature demonstrating the power of aptamer-nanoparticles conjugation for the detection and treatment of cancer. Such conjugates have been used to collect and image leukemia cells [30], detect cancer cells in whole blood [31], create a “super sandwich” cytosensor of aptamer-quantum dots to detect cancer cells [32], target and deliver daunorubicin to leukemia cells [33], and target CEM cells for controlled drug delivery [34]. The present study extends this literature by determining the amount of aptamer on nanoparticles, showing sensitive and specific detection of CCRF-CEM cells over Ramos cells, and evaluating the in vivo toxicity of Sgc8-FSNPs.

## Conclusions

In summary, we have designed a simple, highly sensitive, and specific aptamer-modified FSNPs system for detecting leukemia cells. The usefulness of this system lies in the strong, specific target binding by the aptamer, as well as the fluorescence signal amplification due to the high density of FITC fluorophore in the nanoparticles. This system may prove useful not only for the diagnosis of leukemia but also for the targeted delivery of anti-leukemia drugs. In addition, it may be adaptable to other types of cancer through the selection of appropriate aptamers.

## Abbreviations

APTMS: 3-aminopropylmethyldimethoxysilane
EDC: 1-ethyl-3-(3-dimethylaminopropyl)carbodiimide hydrochloride
FITC: fluorescein isothiocyanate
FSNPs: fluorescent silica nanoparticles
FT-IR: Fourier transform infrared
NH_4_OH: ammonium hydroxide
Sgc8: Sc8 aptamer
sulfo-NHS: *N*-hydroxysulfosuccinimide sodium salt
TEM: transmission electron microscopy
TEOS: tetraethyl orthosilicate
UV–vis: ultraviolet–visible
